# Disseminated *Cryptococcus neoformans* infection associated to COVID-19

**DOI:** 10.1016/j.mmcr.2021.10.001

**Published:** 2021-10-22

**Authors:** Diana Alegre-González, Sabina Herrera, Javier Bernal, Alex Soriano, Marta Bodro

**Affiliations:** a Department of Internal Medicine, Hospital San Pedro, Logroño, 26007, Spain; b Department of Infectious Diseases, Hospital Clinic, Barcelona, 08036, Spain

**Keywords:** COVID-19, Cryptococcus neoformans, Corticosteroids

## Abstract

Severe Acute Respiratory Syndrome Coronavirus 2 (SARS-CoV-2) is a novel coronavirus associated with immune dysregulation. The use of immunosuppressant drugs as part of COVID-19 treatment (such as Tocilizumab or high -dose corticosteroids) increases the risk of opportunistic infections. Here we present a case of a patient without prior immunosuppression that developed a serious fungal infection after the use of high dose corticosteroids in the setting of severe COVID-19 and cryptogenic organizing pneumonia.

## 1 Introduction

The spread of Severe Acute Respiratory Syndrome Coronavirus 2 (SARS-CoV-2) has led to a world pandemic. A heterogenous range of clinical manifestations known as Coronavirus Disease 2019 (COVID-19) has been described, mainly bilateral pneumonia and acute respiratory syndrome caused by a pro-inflammatory state [1]. Severe COVID-19 disease is associated with an increase in pro-inflammatory markers, predominantly IL-1, IL-6, and alpha tumor necrosis factor, together with decreased CD4 and CD8 cells. This phenomenon has been associated with an increased susceptibility to bacterial and fungal infections. Furthermore, the use of immunosuppressant drugs, like IL-6 modulators or high dose corticosteroids, commonly used in COVID-19, increase the risk of opportunistic infections [2].

There have been numerous reports of fungal infections associated to COVID-19, being the most frequent COVID-19 associated pulmonary aspergillosis (CAPA) [3], mucormycosis [4], candidiasis and *Pneumocystis jiroveci* [5]. However, to date only two cases of COVID-19 associated *Cryptococcus neoformans* have been published [6,7].

## 2 Case

A 78 year old man with past history of type 2 diabetes mellitus, high blood pressure and chronic kidney disease (CKD-EPI 34.33 ml/min) was admitted to the emergency department.

**Fig. 1:**
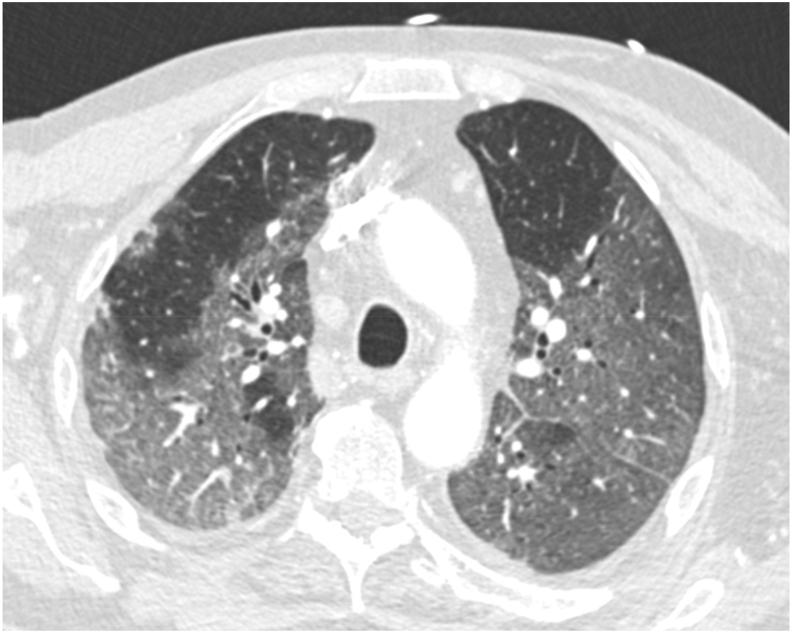
A chest CT showing bilateral ground-glass opacities suggestive of COVID-19.

The patient presented with one week of severe asthenia, dyspnea and fever. On physical examination the oxygen saturation was less than 80% on room air and he presented tachypnea (33 bpm), temperature of 38 °C and crackles in both lungs. Arterial blood gas on room air revealed: pH 7.20 (normal range 7.35–7.45), PaCo2 24 mmHg (NR 35–45 mmHg), PaO2 66 mmHg (NR 75–100 mmHg). Laboratory data revealed leukocyte count 14.2 10^9/L (NR between 4.00 and 11.00), total neutrophil count 13.3 10^9/L (2.0–7.0) total lymphocyte count 0.3 10^9/L (0.9–4.5), CRP 28.87 mg/dL (NR < 1.00), Ferritin 7288 ng/mL (20–400) and LDH 600 U/L (NR < 234). A chest x-ray showed bilateral pulmonary infiltrates. A chest computed tomography scan showed (Fig. 1) bilateral ground-glass opacities suggestive of COVID-19. A nasopharyngeal swab was performed confirming SARS-CoV-2 infection. The patient had high oxygen requirements and was transferred to the ICU. On day +1 treatment with non-invasive mechanical ventilation and high dose corticosteroids (dexamethasone 6 mg/24 hours) was initiated. On day +5 the patient's condition worsened and high oxygen flow therapy was initiated. On day +30 a progressive improvement allowed to reduced the oxygen flow rate to non invasive ventilation with venturi mask (FiO2 42%). On day +58 because of persistent bilateral infiltrates and high oxygen requirements a new chest tomography was performed. It showed cryptogenic organizing pneumonia and treatment with pulse methylprednisolone 250 mg IV (3 days) was started, and thereafter 0.5mg/kg/day. The patient had a favourable evolution and was eventually transferred to a conventional ward and subsequently discharged to a rehabilitation facility on day +62. On discharge the patient was on a 30-day prednisone tapering regimen.

On day +65 the patient was again admitted with fever. Physical exam was unremarkable, with the exception of crackles in both lungs. Laboratory data revealed leukocytes 10.43 10^9/L (4.00–11.00), total neutrophil count 9.9 10^9/L (NR 2.0–7.0) total lymphocyte count 0.2 10^9/L (NR 0.9–4.5), Hemoglobin 89 g/L (NR between 130 and 170), Creatinine 1,55 mg/dL (NR 0.30–1.30), CRP 9.86 mg/dL (NR < 1.00) and procalcitonin 0.37 ng/mL (NR < 0,5). Urine and blood cultures were taken. A new chest X ray showed bilateral infiltrates and a new left consolidation. Empirical treatment with Meropenem (1 gr/12h) and Linezolid (600 mg/12h) was started under the suspicion of nosocomial pneumonia.

**Fig. 2:**
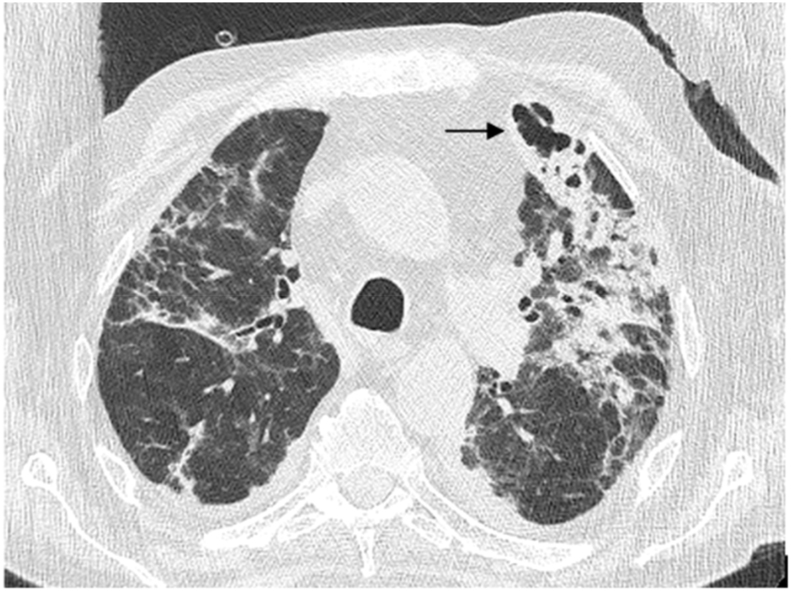
A chest CT showing cryptogenic organizing pneumonia and a subpleural cavitated lesion in the left upper lobe (black arrow).

On day +75 *Cryptococcus neoformans* was isolated from blood cultures. HIV infection was ruled out. A lumbar puncture was performed to rule out CNS involvement, showing: protein 1313 mg/mL (NR 150–450 mg/mL), glucose 91 mg/dL (NR 40–80 mg/dL) with serum glucose 178 mg/dL (NR 65–100 mg/dL) and 0 cells. The opening pressure was 80 mmHg (70–180 mmH2O). CSF cryptococcal antigen (CrAg) was positive at 1/1024 and serum CrAg was 1/512. CSF cultures also yielded *Cryptococcus neoformans.* A CT chest revealed a subpleural cavitated lesion in the left upper lobe (Fig. 2). Treatment was started with Liposomal Amphotericin B (3 mg/Kg/24h) and Flucytosine (25/mg/kg/6h). A new lumbar puncture was performed revealing CSF CrAg of *1/64.* On day +85 he was switched to fluconazole 400 mg/24h due to increasing creatinine levels.

The patient had a favourable evolution in the next few days despite worsering of his general condition, and on day +90 was discharged to a rehabilitation facility. On day +105 he was readmitted again with pneumonia. Meticillin-resistant *Staphylococcus aureus* was isolated from bloodcultures (they were negative for cryptococcus). He was treated with Linezolid 600 mg/12h 14 days. A control CT chest showed improvement of the subpleural cavitated lesion. Treatment with Fluconazole (400mg/24h) was continued. The patient was discharged to a rehabilitation facility on day +128. Unfortunately, the patient died of a new respiratory infection on day +145 at the rehabilitation facility.

## 3 Discussion

*Cryptococcus neoformans* is an encapsulated basidiomycetous. The fungus enters the respiratory tract by inhalation of basidiospores. In immunocompetent individuals it can colonize the respiratory tract without causing any disease. It usually causes infection in immunocompromised individuals. Identified risk factors are: HIV infection, liver cirrhosis and solid organ transplantation [6].

Two other cases of *Cryptococcus neoformans* associated to COVID-19 have been published to the date [6,7]. In the case reported by Passarelli VC et al., the patient had a past history of kidney transplant and liver cirrhosis, and was considered immunosupressed. He also presented with lymphopenia. This immunosuppressive status promoted poor COVID-19 evolution and increased the risk of disseminated cryptococcosis. In the case reported by Khatib MY et al. the patient had no previous history of immunosuppression, similar to our case. The immune dysregulation associated with COVID-19 and the use of immunosuppressant drugs probably triggered *Cryptococcus neoformans* infection. Both patients died a few days after developing Cryptococcemia, similar to our case.

Our patient did not have any neurological manifestations, but CSF showed CNS involvement, without pleocytosis. This phenomenon is probably due to the high degree of immunosuppression, it has been described in the HIV population and it is associated with worse outcomes [8,9] In the case reported by Passarelli VC et al., the diagnosis of cryptococcosis was made post-mortem and therefore CNS involvement was not ruled out. In the case reported by Khatib CNS involvement was not ruled out due to poor prognosis and risk of bleeding.

Here we present a case of a patient without prior immunosuppression that developed a serious fungal infection after the use of high dose corticosteroids in the setting of severe COVID-19 and cryptogenic organizing pneumonia. Imaging can be aspecific and frequently mistaken by infections caused by more common fungal pathogens such as *Aspergillus* sp. Moreover, the patient had CNS involvement without any manifestations. It is important to keep *Cryptococcus neoformans* in the differential diagnosis of COVID-19 associated co-infections, as it has a very high mortality, most notably in patients that present with Cryptococcemia [10].

This report highlights the importance of the risk profile of severe COVID-19 patients for fungal co-infections including disseminated cryptococcosis.

## Conflict of interest

There are none.
